# Investigation of the presence of human adenovirus, norovirus, and rotavirus in tap water in Marrakech, Morocco

**DOI:** 10.1128/aem.01595-25

**Published:** 2026-02-27

**Authors:** Nouhaila Elfellaki, Salma Berrouch, Houda Rafi, Simeon Goïta, Hibatallah Lachkar, Jamal Eddine Hafid

**Affiliations:** 1 Laboratory of Bioresources and Food Safety, Faculty of Sciences and Technology, Cadi Ayyad University, Marrakech, Morocco; 2 Higher School of Technology of El Kelâa des Sraghna, Cadi Ayyad University, Marrakech, Morocco; Michigan State University, East Lansing, Michigan, USA

**Keywords:** Tap water, Marrakech, human adenovirus, human norovirus, rotavirus, RT-qPCR, qPCR detection

## Abstract

Given the significant role played by human pathogenic viruses in causing waterborne diseases, the necessity of monitoring viral contamination in drinking water is paramount for public health. The prevalence of enteric viruses in drinking water remains under-researched in Morocco, with a paucity of data on the issue. The present study, therefore, sought to assess the presence of enteric viruses, namely, human adenovirus (HAdV), human norovirus genogroup I (HNoV-GI) and genogroup II (HNoV-GII), and group A rotavirus (RVA) in tap water in Marrakech. A total of 102 tap water samples were collected, with virus concentration carried out using an internally validated method based on electronegative membranes. The application of quantitative PCR and reverse transcription-quantitative PCR techniques enabled the detection of HAdV in 92.15% of the samples, HNoV-GII in 48.03%, and RVA in 43.13%. It is noteworthy that HNoV-GI was not detected in any of the samples. The results were confirmed by agarose gel electrophoresis. This study is the first of its kind in the region, providing valuable insights into viral contamination in drinking water in Marrakech. In conclusion, the high prevalence of enteric viruses in tap water underscores the need for improved monitoring and more effective water treatment in Marrakech. These findings highlight the importance of prioritizing waterborne virus surveillance to protect public health, with further research needed to investigate viral integrity and infectivity to assess the real risk encountered by consumers.

**IMPORTANCE:**

This study highlights the value of virological monitoring of drinking water as a complementary tool to understand the circulation of enteric viruses in the community. As the first investigation of its kind in Marrakech, it provides essential baseline data in a context where such information is scarce in Morocco. The high prevalence of human adenovirus (92.15%) and the detection of human norovirus genogroup II and group A rotavirus underscore potential public health risks, especially for vulnerable populations. These findings reveal the limitations of relying solely on bacteriological indicators and emphasize the need to integrate routine viral surveillance into water quality monitoring programs. The results also provide scientific evidence to improve treatment and disinfection processes and to support further research on the infectivity of detected viruses.

## INTRODUCTION

Access to safe and easily accessible drinking water is essential for public health. Its availability depends on existing water resources and is strongly influenced by climate change, population growth, social transformations, and urbanization. The World Health Organization (WHO) (1) reported that in 2020, 5.8 billion people had access to drinking water, meeting essential criteria: protection from pollution, availability on-site, accessibility when needed, and absence of contamination, while two billion people still lacked these conditions and were therefore at risk of exposure to unsafe water. Even treated drinking water may pose health risks due to potential pollution that can compromise its quality. Between 1971 and 2006, 780 outbreaks linked to contaminated drinking water were reported in the United States, affecting 577,094 people (2). In Europe, numerous episodes have also been documented; for instance, Spain reported 413 outbreaks between 1999 and 2006, affecting 23,642 individuals (3). Of the 117 outbreaks with a confirmed etiology, most (95.72%) were caused by infectious agents. Among the 48 viral outbreaks, norovirus was the most frequently detected pathogen (54.16%), followed by hepatitis A virus (29.16%) and rotavirus (12.5%) (3).

Enteric viruses are therefore a major concern for public health due to their ability to cause widespread infections (1). Human adenovirus (HAdV) is recognized as a potential contaminant of drinking water (4), while human noroviruses (HNoVs) are responsible for millions of cases of non-bacterial acute gastroenteritis worldwide (5). Group A rotavirus (RVA), the leading cause of severe gastroenteritis in infants, remains a significant contributor to child morbidity and mortality (6, 7). Following WHO recommendations, the Ministry of Health of Morocco introduced the monovalent rotavirus vaccine, Rotarix, into the national vaccination program in 2010 to reduce the high burden of rotavirus disease in the country (8).

This underscores the imperative for vigilant monitoring and control of pathogens in water, especially in regions exhibiting high vulnerability.

The presence of these viruses in drinking water remains a significant public health concern, as even at low concentrations, they can cause illness after ingestion (9). In this context, numerous techniques have been developed since the 1960s for various types of water (10). Among these approaches, the use of a magnesium chloride (MgCl_2_) pretreatment followed by filtration through electronegative membranes, combined with molecular methods, has emerged as a commonly employed strategy (11–19). These methodological advancements signify a pivotal stage in the evaluation of the viral risks associated with drinking water. In a previous stage of this study, several concentration methods were evaluated. Among these, a method based on preconditioning with MgCl_2_, followed by filtration through an electronegative membrane and elution with sodium hydroxide, proved to be effective for the simultaneous recovery of HAdV, human norovirus genogroup I (HNoV-GI)/genogroup II (HNoV-GII), and RVA from tap water (20).

Globally, numerous studies have focused on the detection of enteric viruses in water samples, covering various types of water sources. A meta-analysis revealed a global prevalence of AdV in water of 59.28% (95% confidence interval [CI]: 54.46–64.02), with variations depending on the water matrix; for drinking water, the prevalence was 22.70% (95% CI: 12.61–34.53) (21). HNoV has also been reported worldwide, with a prevalence of 31.7% (95% CI: 25.1–38.5), although this varied according to the type of water source. The meta-analysis indicated the highest HNoV prevalence in Africa, at 55.9% (95% CI: 28.2–81.9) (22). For RVA, another meta-analysis estimated the global prevalence of RVA in water environments at 40.86% (95% CI: 34.04–47.85). The prevalence in drinking water was lower, at 9.46% (95% CI: 3.71–17.18). In Africa, the prevalence was notably higher, reaching 51.75% (23).

In Morocco, no studies to date have specifically addressed the prevalence of enteric viruses in surface waters or in water intended for human consumption. Existing research has primarily focused on the detection of these viruses in marine environments, particularly in mussels and other shellfish, which are known bioaccumulators of waterborne pathogens (24, 25).

In this context, an in-depth study was conducted to assess water quality and detect the presence of HAdV, HNoV-GI/HNoV-GII, and RVA in Marrakech by applying a sensitive concentration method in water, using quantitative real-time PCR and RT-PCR.

## MATERIALS AND METHODS

### Sample collection

A total of 102 2 L samples were collected over a 1-year period, from February 2024 to February 2025, except in August 2024, with two samples collected per week from the laboratory of Bioresources and Food Safety at the Faculty of Science and Technology in Marrakech. The laboratory is supplied by the municipal water treatment plant managed by the National Office of Electricity and Potable Water, which relies on 93% surface water and 7% groundwater as its sources. It applies a conventional treatment procedure including coagulation–flocculation, sedimentation, sand filtration, and disinfection by chlorination.

The collected samples were stored at 4°C, filtered, and analyzed within 24 h of sampling.

### Physicochemical parameters of water samples

In order to assess water quality and identify potential PCR inhibitors, we measured physicochemical parameters, namely, temperature, pH, turbidity, conductivity, and dissolved oxygen, using a portable analysis kit (multi-parameter portable meter MultiLine).

### Virus concentration method

The water samples were processed using an adsorption–elution method with membrane filtration, as previously described (20). First, anhydrous MgCl_2_ was added to the water samples to achieve a final concentration of 25 mM. This created the necessary conditions to promote the adsorption of viruses onto the negatively charged membranes. The samples were then filtered through negatively charged nitrocellulose membranes (47 mm diameter, 0.45 μm pore size; Sartorius). This type of membrane is commonly used to trap viruses in water samples. To remove any remaining cations on the membrane, 200 mL of a 0.5 mM H_2_SO_4_ solution (pH 3.0) was passed through the filter. This step is crucial for neutralizing the cation charges and facilitating the release of the captured viruses. Next, the virus elution was performed by adding 5 mL of 1.0 mM NaOH (pH 10.8), which alters the pH of the solution and allows the viruses to be eluted from the membrane. The solution was agitated at 120 rpm for 30 min, followed by three 1-min vortex cycles to ensure uniform distribution of the viruses in the solution. The resulting supernatant was collected in a tube containing 50 μL of 100 mM H_2_SO_4_ and 100 μL of 100 mM Tris-EDTA, which serve as neutralization buffers to stabilize the viruses. Finally, the supernatant was centrifuged at high speed (10,000 × *g*) for 45 min to concentrate the virus by reducing the volume to approximately 1 mL. A second centrifugation step was performed at the same speed for 30 min to achieve a final volume of 150 µL.

### Positive and negative controls

To ensure the reliability of the analyses, positive and negative controls were prepared. Separate 2 L water samples were heated to 100°C for 30 min and then allowed to cool. After cooling, each sample was spiked with 30 µL of viral suspension containing either HAdV, HNoV-GI, HNoV-GII, or RVA. Viral suspensions were obtained from positive diarrheic stool samples of infected patients, generously provided by the virology laboratories of the University Hospitals of Dijon and Saint-Étienne, France. To determine the quantification cycle (Cq) values for each sample in our laboratory, 30 μL of stool was added directly, without any prior preparation, to 120 μL of ultrapure water. This mixture, with a final volume of 150 μL, was used for nucleic acid extraction. The extracted nucleic acid was then amplified by real-time PCR and RT-PCR. The spiked water samples were homogenized by stirring to ensure even virus distribution before analysis. These samples served as positive controls, essential for detecting potential false negatives. A sterilized, non-spiked sample was used as a negative control to confirm the absence of cross-contamination.

### Real-time PCR and real-time RT-PCR settings

Nucleic acid extraction was performed using NucleoSpin RNA columns (Macherey-Nagel), following the manufacturer’s instructions. Nucleic acids were eluted in a final volume of 80 μL. PCR conditions were set as described below (Table 1).

**TABLE 1:** Target gene, primer/probe sequences, and amplification conditions for real-time PCR and real-time RT-PCR assays used in this study^*a*^

Assay	Target gene	Primer or probe sequence (5′–3′)	Amplification conditions	Reference(s)
HAdV qPCR	Hexon gene	P: FAM-CCGGGCTCAGGTACTCCGAGGCGTCCT-BHQ F: C(AT)TACATGCACATC(GT)C(CG)GG R: C(AG)CGGGC(GA)AA(CT)TGCACCAG	3 min at 95°C 40 cycles of 15 s at 95°C and 30 s at 60°C	(26)
HNoV-GII RT-qPCR	ORF2	P: FAM-AGC ACG TGG GAG GGC GAT CG-TAMRA F: ATG TTC AGR TGG ATG AGR TTC TCW GA R: TCG ACG CCA TCT TCA TTC ACA	30 min at 50°C 5 min at 95°C 45 cycles of 10 s at 95°C and 20 s at 55°C	(27–30)
HNoV-GI RT-qPCR	Capsid	P: FAM- TGGACAGGAGAYCGCRATCT-TAMRA F: CGCTGGATGCGNTTCCAT R: CCTTAGACGCCATCATCATTTAC	30 min at 50°C 5 min at 95°C 45 cycles of 10 s at 95°C and 20 s at 55°	(28, 31)
RVA RT-qPCR	Gene NSP3	P: FAM-AGTTAAAAGCTAACACTGTCAAA-TAMRA F: ACCATCTWCACRTRACCCTCTATGAG R: GGTCACATAACGCCCCTATAGC	30 min at 48°C 10 min at 95°C 40 cycles of 15 s at 95°C and 60 s at 60°C	(32, 33)

^*a*^ BHQ, black hole quencher; F, forward primer; FAM, 6-carboxyfluorescein; P, probe; qPCR, quantitative PCR; R, reverse primer; RT-qPCR, reverse transcription-quantitative PCR; TAMRA, 6-carboxytetramethylrhodamine.

The PCR mixtures for the detection of HAdV and HNoV-GII were prepared as previously described in reference 20.

For HNoV-GI, the same one-step RT-PCR mixture as for HNoV-GII was used, with the Sensifast Probe Lo-RoX One-Step kit (Meridian Bioscience) in a 20 μL reaction mixture, which included 4 μL of RNA templates, 16 μL of master mix containing 400 nM of each primer, 100 nM of probe, and RNase-free water up to the final volume.

For RVA detection, the RNA extracts were denatured for 5 min at 97°C, followed by cooling on ice for 5 min to separate the double-stranded RNA. Real-time RT-PCR assays were performed using the 4× CAPITAL 1-Step qRT-PCR Probe Master Mix (biotechrabbit) in a 20 μL reaction mixture containing 5 μL of RNA templates, 15 μL of master mix with 400 nM of each primer, 100 nM of probe, and RNase-free water up to the final volume.

All PCR analyses were performed using a MyGo Pro Real-Time PCR instrument. PCR reactions were conducted in duplicate, and the Cq values were averaged (Cq ≤40 were considered positive).

For each water sample, 10 µL of PCR product was collected and analyzed as follows.

### Gel electrophoresis analysis

**Fig 1:**
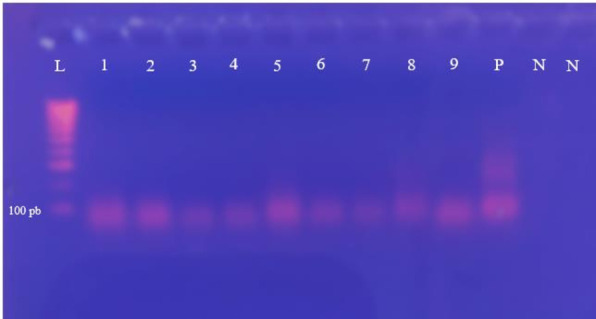
Visualization of PCR products for HAdV-positive tap water samples by gel electrophoresis. Lane L,100 bp marker; Lane P, positive control; Lane N, negative control; Lanes 1, 2, 3, 4, 5, 6, 7, 8, and 9, positive tap water samples for HAdV.

PCR products were analyzed by a 2% agarose gel electrophoresis containing 5 µL of ethidium bromide (0.3 µg/mL). Each sample was mixed with 2 µL of loading dye (biotechrabbit) and loaded onto the gel alongside a 100 bp molecular weight marker (DNA Ladder with 6× Loading Dye, biotechrabbit) and positive and negative controls. The gel was then immersed in 1× TBE buffer (Tris-borate-EDTA, pH 8.0) and electrophoresed at 120 V for 40–45 min. After electrophoresis, the amplified products were visualized under UV light using a UV transilluminator following ethidium bromide staining. Amplicon sizes were determined by comparison with the molecular weight marker and controls (Fig. 1).

### Statistical analysis

All statistical analyses were performed using R software (version 4.4.1). The impact of seasonality on the prevalence of each virus was assessed using Fisher’s exact test.

To determine whether the prevalence was influenced by physicochemical parameters (temperature, pH, dissolved oxygen, conductivity, and turbidity), Mann–Whitney tests were conducted to compare the distribution of each parameter between virus-positive and virus-negative samples.

## RESULTS

### Physicochemical parameters of water samples

The physicochemical properties of the majority of water samples collected across a year demonstrated overall stability, except for temperature and conductivity. Recorded pH averaged 7.53 ± 0.25 (7.03–8.01), indicating near-neutral to slightly alkaline conditions. Water temperature averaged 20.70°C ± 5.55°C (11.5 °C–37 °C), reflecting seasonal variation. The average electrical conductivity was 940.62 ± 419.22 μS/cm. The high variation may reflect a large variability between samples, likely due to seasonal variation and water supply interruptions during the sampling period. Dissolved oxygen (DO) levels averaged 8.76 ± 0.20 mg/L. Turbidity values remained low in all samples, with a mean value of 1.03 ± 0.53 nephelometric turbidity unit (NTU), pointing to limited particulate matter and overall water clarity (Table 2).

**TABLE 2:** Physicochemical parameters of tap water samples^*a*^

Physicochemical parameter	Overall mean ± SD
Temperature (**°**C)	20.71 ± 5.55
pH	7.53 ± 0.25
Conductivity (µS/cm)	947.50 ± 415.41
Turbidity (NTU)	1.03 ± 0.53
Dissolved oxygen (mg/L)	8.76 ± 0.20

^*a*^ NTU, nephelometric turbidity unit; SD, standard deviation.

### Study limitations

This study has a few limitations. First, the viral concentration in the samples could not be determined, as quantitative standards were not included, and the limit of detection of the method has not yet been established. In addition, the detection relied on molecular assays, which indicate the presence of viral genomes but do not confirm infectivity. Finally, the sampling strategy may not fully capture the spatial and temporal variability of viral contamination.

### Detection of enteric viruses in drinking water samples

PCR and RT-PCR analysis of 102 tap water samples revealed a high occurrence of enteric viruses throughout the year. HAdV was the most frequently detected, with a prevalence of 92.15% (94/102), followed by HNoV-GII at 48.03% (49/102) and RVA at 43.13% (44/102). Notably, NoV-GI was not detected in any of the samples throughout the year. A total of 31.37% (32/102) of the samples were simultaneously positive for all three viruses tested (HAdV, HNoV-GII, and RVA); 10.78% (11/102) were co-positive for HAdV and HNoV-GII; 5.88% (6/102) were co-positive for HNoV-GII and RVA; and 4.90% (5/102) were co-positive for HAdV and RVA (Table 3). All positive samples detected by real-time PCR and RT-PCR were subjected to gel electrophoresis analysis. This method confirmed the results by clearly visualizing the migrated DNA fragments, validating the presence of the viral genome.

**TABLE 3:** Monthly distribution of samples positive for one, two, or three viruses simultaneously from February 2024 to February 2025 in Marrakech

Sample date (mo-yr)	HAdV	HAdV/Hu-NoV:GII	HAdV/RVA	Hu-NoV:GII	Hu-NoV:GII/RVA	RVA	HAdV/Hu-NoV:GII/RVA	Total positive samples
Feb-24	0/9	0/9	0/9	0/9	0/9	0/9	9/9	9/9
Mar-24	0/7	0/7	1/7	0/7	0/7	0/7	6/7	7/7
Apr-24	0/9	0/9	0/9	0/9	1/9	0/9	8/9	9/9
May-24	1/9	6/9	0/9	0/9	0/9	0/9	2/9	9/9
June-24	0/8	2/8	3/8	0/8	0/8	0/8	3/8	8/8
July-24	5/8	3/8	0/8	0/8	0/8	0/8	0/8	8/8
Sept-24	9/9	0/9	0/9	0/9	0/9	0/9	0/9	9/9
Oct-24	9/9	0/9	0/9	0/9	0/9	0/9	0/9	9/9
Nov-24	8/8	0/8	0/8	0/8	0/8	0/8	0/8	8/8
Dec-24	8/9	0/9	0/9	0/9	0/9	0/9	0/9	8/9
Jan-25	6/9	0/9	1/9	0/9	1/9	0/9	1/9	9/9
Feb-25	0/8	0/8	0/8	0/8	4/8	1/8	3/8	8/8
Total	46/102	11/102	5/102	0/102	6/102	1/102	32/102	101/102

The distribution of positive water samples varied by season (Table 4). HAdV was detected consistently throughout the year, indicating a persistent environmental presence independent of seasonal variation. Lower mean Cq values in winter (28.91 ± 1.20) and spring (29.26 ± 4.54) suggest higher viral loads during these periods. In contrast, HNoV-GII and RVA displayed clear seasonality, with the highest detection rates observed in winter (28.82 ± 2.42 and 30.31 ± 1.11, respectively) and spring (25.63 ± 1.74 and 29.50 ± 0.91), and complete absence in autumn. However, the Cq values for RVA remained relatively stable across seasons, indicating that viral concentrations, when present, were relatively consistent. These findings highlighted the seasonal dynamics of HNoV-GII and RVA, in contrast to the more constant year-round detection of HAdV, which may reflect differences in environmental stability or transmission routes.

**TABLE 4:** Seasonal distribution of positive tap water samples for HAdV, HNoV-GII, and RVA from Winter 2024 to Winter 2025 in Marrakech^*a*^

Virus	No. of positive samples/total analyzed samples (mean Cq ± SD)					Total positive samples/total analyzed samples
	Winter 2024	Spring 2024	Summer 2024	Autumn 2024	Winter 2025	
HAdV	14/14 (28.91 ± 1.20)	25/26 (29.26 ± 4.54)	16/16 (35.94 ± 0.51)	25/26 (35.99 ± 0.70)	14/20 (35.62 ± 0.58)	94/102
HNoV-GII	13/14 (28.82 ± 2.42)	24/26 (25.63 ± 1.74)	3/16 (30.33 ± 1.79)	0/26	9/20 (26.17 ± 1.85)	49/102
RVA	14/14 (30.31 ± 1.11)	17/26 (29.50 ± 0.91)	2/16 (28.18 ± 0.18)	0/26	11/20 (31.19 ± 1.10)	44/102

^*a*^ Cq, quantification cycle; SD, standard deviation.

The influence of seasonality on the presence of viruses was analyzed between February 2024 and February 2025, using the exact Fisher’s test. No significant seasonal effect was observed for HAdV (*P* = 0.1267). In contrast, a statistically significant association was found between seasonality and the presence of Hu-NoV-GII and RVA (*P* < 0.0001).

The Mann–Whitney test revealed a significant association between certain physicochemical parameters and the presence of the investigated viruses (Table 5). Temperature significantly affected the presence of all three viruses (*P* < 0.05). Additionally, pH influenced the presence of RVA (*P* < 0.05) but had no effect on HAdV or HNoV-GII. Conductivity was also significantly associated with the detection of both HNoV-GII and RVA (*P* < 0.001), unlike HAdV. In contrast, no statistically significant associations were detected between turbidity or dissolved oxygen and the presence of any of the three viruses (*P* > 0.05).

**TABLE 5:** Results of the Mann–Whitney tests assessing the influence of physicochemical parameters on the presence of the three viruses^*a*^

Physicochemical parameter	HAdV	HNoV-GII	RVA
Temperature (**°**C)			
Mean (+)	21.11 ± 5.57	19.44 ± 4.87	18.11 ± 3.83
Mean (−)	16.00 ± 2.39	21.88 ± 5.92	22.68 ± 5.87
*P* value	0.010	0.040	<0.001
pH			
Mean (+)	7.52 ± 0.24	7.57 ± 0.25	7.59 ± 0.23
Mean (−)	7.62 ± 0.31	7.49 ± 0.24	7.49 ± 0.25
*P* value	0.337	0.113	0.035
Conductivity (µS/cm)			
Mean (+)	948.60 ± 424.73	643.14 ± 374.51	607.55 ± 397.11
Mean (−)	934.62 ± 304.95	1,228.89 ± 194.22	1,205.40 ± 172.98
*P* value	0.407	<0.001	<0.001
Turbidity (NTU)			
Mean (+)	1.03 ± 0.54	1.07 ± 0.59	1.05 ± 0.57
Mean (−)	1.08 ± 0.40	1.00 ± 0.46	1.02 ± 0.49
p-value	0.328	0.707	0.727
Dissolved oxygen (mg/L)			
Mean (+)	8.76 ± 0.20	8.75 ± 0.22	8.78 ± 0.22
Mean (−)	8.71 ± 0.18	8.77 ± 0.18	8.74 ± 0.19
*P* value	0.403	0.709	0.270

^*a*^ Mean (+), mean value of physicochemical parameter for virus-positive samples; mean (−), mean value of physicochemical parameter for virus-negative samples; NTU, nephelometric turbidity unit; SD, standard deviation.

## DISCUSSION

Surface waters used for drinking water production may contain human pathogenic viruses, which may affect the quality of water intended for consumption. In this study, we evaluated the quality of tap water distributed in Marrakech, Morocco. This is the first report on the presence of HAdV, HNoV GI/HNoV GII, and RVA in tap water, the main source of drinking water in the city. At least one virus was detected in 99% of the analyzed samples. Such results were higher than those generally reported in previous studies (14, 23, 27).

In the literature, few investigations have focused specifically on tap water, although in most works, the term “treated drinking water” consistently refers to potable water that has undergone full treatment at the drinking water treatment plant and is subsequently distributed to households through the municipal network (i.e., tap water). This corresponds exactly to the type of water analyzed in our study. Reported prevalence worldwide nevertheless varies considerably.

For instance, for HAdV, our detection rate, which reached 92.15%, was in the same range as that observed in China, where 100% (24/24) of treated drinking water samples collected from six treatment plants were positive (34). In contrast, in other African countries such as Egypt, a lower prevalence was reported (33%, 8/24) in treated water samples (35), and only (8.9%, 16/1,800) in tap water samples (36). In other countries, such as Pakistan, AdV was reported in 38.94% of drinking water samples (37/95) (37) and 20% of treated water (6/30) (19).

Regarding HNoV-GII, our detection rate of 48.03% was lower than or comparable to that reported in certain countries such as Japan, where rates ranged from 48% to 81% during epidemic periods and from 10% to 24% outside these periods in samples from drinking water treatment plants (38). However, it was higher than those reported in other countries, such as Italy (20%, 1/5) (39), Brazil (7.7%, 2/26) (40), and Slovenia (0/89) (41).

Similarly, the non-detection of HNoV-GI has been reported in several studies on drinking water, such as that in reference 42. This may partly explain the lack of research on this genogroup in these matrices, especially when compared to genogroup II.

Regarding RVA, our study revealed a detection rate of 43.13%, which remained lower than those observed in other geographical contexts. In Japan, for example, rates ranged from 76% to 86% during epidemic periods, and from 67% to 81% during non-epidemic periods in samples collected from drinking water treatment plants (38). In China, 100% of treated water samples collected from six treatment plants tested positive (34). Conversely, lower rates have been reported in other countries: 23% in treated water in Pakistan (7/30) (19), 15.6% in tap water in Egypt (28/100) (36), 7.14% in drinking water in Saudi Arabia (1/14) (43), and only 0.54% in Thailand, also in tap water (2/370) (44).

The variability in virus prevalence rates in water can be attributed to a combination of environmental and social factors, viral structure characteristics, and differences in the technical methods used for water sampling, concentration, and virus detection. Climatic conditions, particularly precipitation, promote the mobilization of fecal contaminants in watersheds, increasing surface water contamination (45). Elevated water flow during these periods may also facilitate the transport of viruses, thereby increasing their detection rates (46). Some enteric viruses, such as Hu-NoV-GII and RVA, exhibit strong seasonal dynamics, with higher prevalence in winter and spring, while HAdV is detected consistently throughout the year due to its environmental stability across a wide range of temperatures (47). Socio-geographical factors, including population density, hygiene practices, and the state of infrastructure, further influence virus prevalence. In developing countries, rapid population growth and inadequate drainage systems exacerbate contamination risks, especially during the rainy season (48).

In addition to environmental and social parameters, differences in technical procedures can also contribute to variability across studies. Reference 45 highlighted that factors such as water quality and sample volume significantly affect recovery and detection efficiency. Physicochemical properties of viruses, including their isoelectric point, influence adsorption behavior and recovery rates. Moreover, reference 49 highlighted the importance of interactions between the virus type and the water matrix, suggesting that factors affecting virus recovery, such as pH during concentration, should be carefully adjusted to optimize detection. As a result, differences in sampling strategies, concentration protocols, and detection methods (e.g., sensitivity of molecular assays) can contribute to variability and discrepancies observed between studies.

The high prevalence of these viruses in tap water in Marrakech could be explained by several interdependent factors. First, this frequent detection was likely due to persistent water contamination, suggesting insufficient water treatment, particularly during the disinfection stage. This stage relies on chlorination for its bactericidal effect, but enteric viruses are more resistant to chlorine inactivation (1). Nevertheless, some studies have demonstrated the effectiveness of combined water treatment systems, which included polypropylene yarn filtration, granular activated carbon, and ultraviolet post-treatment (19). In addition to the quality of water treatment at pumping stations, the water supply system could also be a contributing factor. Leaks in pipes or defective water tanks can provide additional sources of contamination (19). Moreover, rapid population growth and a lack of urban planning place considerable pressure on drainage systems, especially during the rainy season, increasing the risk of surface water contamination (48).

Furthermore, our previous study on the prevalence of parasites in tap water in Marrakech between October 2016 and January 2018 has revealed an overall contamination rate of 67.3% (70/104) for protozoa, with 35 samples positive for *Giardia duodenalis*, 18 for *Toxoplasma gondii*, and 17 for both parasites (50). Our findings highlighted the contamination of water, often of fecal origin, from human or animal sources, indicating the presence of viruses and parasites in drinking water.

Our study revealed a significant seasonal effect on the presence of HNoV-GII and RVA, with higher detection rates in winter and spring, whereas HAdV was consistently detected throughout the year. The increased prevalence of HNoV-GII and RVA during colder months may be explained by the well-documented influence of temperature on viral persistence, as lower temperatures slow viral degradation and enhance environmental stability (51, 52). In contrast, the constant detection of HAdV aligns with its strong environmental resilience, which enables it to remain stable across a broad temperature range (47), a pattern also reported in other studies showing no seasonal variation in HAdV prevalence (51, 52).

Temperature is widely recognized as a major determinant of viral inactivation in the environment (53). High temperatures can alter viral proteins or nucleic acids, accelerating inactivation, while the extent of this effect depends on virus-specific thermal sensitivities and the physicochemical characteristics of the water matrix. These combined factors likely contribute to the observed differences between viruses in their seasonal detection patterns.

pH also played a role in our findings, but its effect was limited to RVA. Viral detection in this study relied on nucleic acid-based assays, meaning that measured “presence” reflects genetic material rather than infectious viral particles. RVA detection was significantly influenced by pH values between 7.03 and 8.01, which is consistent with studies showing that rotaviruses can remain stable in neutral to slightly alkaline conditions, while extreme pH values can inactivate the virus by altering structural proteins (54, 55). This sensitivity may also be modulated by interactions with other water quality parameters, such as organic matter or ion content (56). Additionally, our concentration protocol, which involves pH reduction, addition of multivalent cations, and alkaline elution, may have contributed to variations in RVA recovery. In contrast, HAdV and HNoV-GII are known to withstand a wide pH range (57–59), which may explain the absence of a detectable pH effect and their higher prevalence in our samples. Furthermore, the observed inverse relationship between water conductivity and the detection of HNoV and RVA may be attributed to the physicochemical characteristics of these viruses. Both HNoV and RVA are non-enveloped viruses with capsid surface charges that are sensitive to environmental ionic strength. Studies have shown that HNoV virus-like particles possess isoelectric points (pI) around pH 5.0–6.0, and their net surface charge becomes more negative as the pH increases above the pI. At higher ionic strengths, the electrostatic repulsion between particles is screened, promoting aggregation and adsorption to surfaces, which can reduce their mobility and detectability in water samples (60). Similarly, RVA particles exhibit a negatively charged outer surface, which may enhance their propensity to aggregate or adhere to surfaces under conditions of elevated ionic strength (61).

In contrast, HAdV, particularly enteric types like HAdV-41, displays unique physicochemical properties that confer greater stability across varying ionic conditions. The capsid proteins of HAdV-41 have higher predicted pI values, with the short fiber protein exhibiting a pI of approximately 9.13, indicating a more positive surface charge at neutral pH. This positive charge may reduce the tendency of HAdV-41 to aggregate or adsorb to negatively charged surfaces in high-conductivity environments, thereby maintaining its detectability in water samples (62).

In our study, the mean turbidity of positive and negative samples was approximately 1 NTU, which represents a narrow range of turbidity values. Although higher turbidity levels were typically associated with increased concentrations of organic matter and suspended particles that can adsorb viral particles, potentially affecting their persistence in water (63), the low turbidity variation in our samples may have masked any potential effects.

These combined factors explain the high prevalence of viral contamination in tap water in Marrakech, thus highlighting the public health challenges associated with managing drinking water in rapidly growing urban environments.

To complement molecular detection of viral genetic material, future studies should incorporate methods capable of assessing the actual infectivity of viruses in water. Although PCR and quantitative PCR are widely used due to their speed, sensitivity, and ability to detect viruses that are difficult to culture (64, 65), these approaches do not distinguish infectious from inactivated particles, limiting the accurate assessment of public health risk (66). Recent advances, such as digital PCR, provide absolute quantification and greater tolerance to inhibitors commonly present in environmental samples (67), yet they remain insufficient for evaluating infectivity. Integrated approaches combining cell culture and molecular amplification, such as ICC-PCR, allow for the specific detection of replicating viruses and overcome the limitations of purely culture-based methods (68–70). Additional techniques, including the detection of viral replication intermediates or enzymatic/chemical treatment of viral capsids prior to RT-PCR, can further refine assessments of infectivity (71).

Moreover, expanding sampling across multiple points within the water distribution network would provide a more comprehensive characterization of spatial contamination patterns and a representative assessment of overall water quality. Such an approach would enhance the relevance and applicability of the findings for informed water management and public health interventions.

### Conclusion

Regular surveillance of enteric viruses in drinking water is a critical component in the prevention of waterborne viral infections and potential epidemics. The results of this study highlighted a concerning level of viral contamination, underscoring the need for additional research focusing on viral viability, infectivity, and genotypic diversity to accurately evaluate the health risks faced by exposed populations. Given the potential for exposure through contaminated water, it is imperative to reinforce both treatment protocols and quality control measures throughout the water distribution process.

## References

[1] WHO. 2022. Guidelines for drinking-water quality

[2] Craun GF, Brunkard JM, Yoder JS, Roberts VA, Carpenter J, Wade T, Calderon RL, Roberts JM, Beach MJ, Roy SL. 2010. Causes of outbreaks associated with drinking water in the United States from 1971 to 2006. Clin Microbiol Rev 23:507–528. doi:10.1128/CMR.00077-09 20610821 PMC2901654

[3] Granado AM, Martínez MC, Frías A T, V, Banegas O, Sánchez EV. 2008. Red Nacional de Vigilancia epidemiológica. Vigilancia epidemiológica de brotes de transmisión hídrica en españa. 1999-2006. Bol epidemiol Semanal 16

[4] Jiang SC. 2006. Human adenoviruses in water: occurrence and health implications: a critical review. Environ Sci Technol 40:7132–7140. doi:10.1021/es060892o 17180959

[5] Glass RI, Parashar UD, Estes MK. 2009. Norovirus gastroenteritis. N Engl J Med 361:1776–1785. doi:10.1056/NEJMra0804575 19864676 PMC3880795

[6] Parashar UD, Gibson CJ, Bresee JS, Glass RI. 2006. Rotavirus and severe childhood diarrhea. Emerg Infect Dis 12:304–306. doi:10.3201/eid1202.050006 16494759 PMC3373114

[7] Sanchez-Padilla E, Grais RF, Guerin PJ, Steele AD, Burny M-E, Luquero FJ. 2009. Burden of disease and circulating serotypes of rotavirus infection in sub-Saharan Africa: systematic review and meta-analysis. Lancet Infect Dis 9:567–576. doi:10.1016/S1473-3099(09)70179-3 19695493

[8] Benhafid M, Rguig A, Trivedi T, Elqazoui M, Teleb N, Mouane N, Maltouf AF, Parashar U, Patel M, Aouad RE. 2012. Monitoring of rotavirus vaccination in Morocco: establishing the baseline burden of rotavirus disease. Vaccine (Auckl) 30:6515–6520. doi:10.1016/j.vaccine.2012.08.058 22959990

[9] Ward RL, Akin EW, D’Alessio DJ. 1984. Minimum infective dose of animal viruses. Critical Reviews in Environmental Control 14:297–310. doi:10.1080/10643388409381721

[10] Bofill-Mas S, Rusiñol M. 2020. Recent trends on methods for the concentration of viruses from water samples. Curr Opin Environ Sci Heal 16:7–13. doi:10.1016/j.coesh.2020.01.006

[11] Katayama H, Shimasaki A, Ohgaki S. 2002. Development of a virus concentration method and its application to detection of enterovirus and norwalk virus from coastal seawater. Appl Environ Microbiol 68:1033–1039. doi:10.1128/AEM.68.3.1033-1039.2002 11872447 PMC123733

[12] Rigotto C, Victoria M, Moresco V, Kolesnikovas CK, Corrêa AA, Souza DSM, Miagostovich MP, Simões CMO, Barardi CRM. 2010. Assessment of adenovirus, hepatitis A virus and rotavirus presence in environmental samples in Florianopolis, South Brazil. J Appl Microbiol 109:1979–1987. doi:10.1111/j.1365-2672.2010.04827.x 20698910

[13] Kishida N, Morita H, Haramoto E, Asami M, Akiba M. 2012. One-year weekly survey of noroviruses and enteric adenoviruses in the Tone River water in Tokyo metropolitan area, Japan. Water Res 46:2905–2910. doi:10.1016/j.watres.2012.03.010 22465727

[14] Fongaro G, Nascimento MA do, Rigotto C, Ritterbusch G, da Silva ADA, Esteves PA, Barardi CRM. 2013. Evaluation and molecular characterization of human adenovirus in drinking water supplies: viral integrity and viability assays. Virol J 10:1–9. doi:10.1186/1743-422X-10-166 23714224 PMC3686584

[15] Spilki FR, Luz RB da, Fabres RB, Soliman MC, Kluge M, Fleck JD, Rodrigues MT, Comerlato J, Cenci A, Cerva C, Dasso MG, Roehe PM. 2013. Detection of human adenovirus, rotavirus and enterovirus in water samples collected on dairy farms from Tenente Portela, Northwest of Rio Grande do Sul, Brazil. Braz J Microbiol 44:953–957. doi:10.1590/S1517-83822013000300046 24516464 PMC3910217

[16] Silverman AI, Akrong MO, Amoah P, Drechsel P, Nelson KL. 2013. Quantification of human norovirus GII, human adenovirus, and fecal indicator organisms in wastewater used for irrigation in Accra, Ghana. J Water Health 11:473–488. doi:10.2166/wh.2013.025 23981876

[17] Gularte JS, Staggemeier R, Demoliner M, Heck TMS, Heldt FH, Ritzel RGF, Rigotto C, Henzel A, Spilki FR. 2017. Human adenovirus in tissues of freshwater snails living in contaminated waters. Environ Monit Assess 189:276. doi:10.1007/s10661-017-5979-2 28523581

[18] Ahmad T, Adnan F, Nadeem M, Kakar SJ, Anjum S, Saad A, Waheed A, Arshad N. 2018. Assessment of the risk for human health of Enterovirus and Hepatitis A virus in clinical and water sources from three metropolitan cities of Pakistan. Ann Agric Environ Med 25:708–713. doi:10.26444/aaem/99590 30586962

[19] Rashid M, Khan MN, Jalbani N. 2021. Detection of human adenovirus, rotavirus, and enterovirus in tap water and their association with the overall quality of water in karachi, pakistan. Food Environ Virol 13:44–52. doi:10.1007/s12560-020-09448-8 33180282

[20] Elfellaki N, Berrouch S, Biary A, Goïta S, Rafi H, Lachkar H, Dehhani O, de Rougemont A, Bourlet T, Hafid JE. 2024. Comparison of four concentration methods of adenovirus, norovirus and rotavirus in tap water. J Virol Methods 330:115013. doi:10.1016/j.jviromet.2024.115013 39209160

[21] Takuissu GR, Kenmoe S, Ebogo-Belobo JT, Kengne-Ndé C, Mbaga DS, Bowo-Ngandji A, Ondigui Ndzie JL, Kenfack-Momo R, Tchatchouang S, Kenfack-Zanguim J, Lontuo Fogang R, Zeuko’o Menkem E, Kame-Ngasse GI, Magoudjou-Pekam JN, Suffredini E, Veneri C, Mancini P, Bonanno Ferraro G, Iaconelli M, Verani M, Federigi I, Carducci A, La Rosa G. 2024. Exploring adenovirus in water environments: a systematic review and meta-analysis. Int J Environ Health Res 34:2504–2516. doi:10.1080/09603123.2023.2255559 37678554

[22] Ekundayo TC, Igere BE, Oluwafemi YD, Iwu CD, Olaniyi OO. 2021. Human norovirus contamination in water sources: a systematic review and meta-analysis. Environ Pollut 291:118164. doi:10.1016/j.envpol.2021.118164 34534825

[23] Awere-Duodu A, Donkor ES. 2024. Rotavirus in water environments: a systematic review and meta-analysis. Environ Health Insights 18. doi:10.1177/11786302241276667 PMC11494518 39439598

[24] Boussettine R, Hassou N, Maanan M, Bessi H, Ennaji MM. 2023. Hepatitis A virus detection by RT-qPCR in shellfish samples from three Moroccan Atlantic coastal areas: Dakhla, Oualidia, and Moulay Bousselham. Lett Appl Microbiol 76:ovac059. doi:10.1093/lambio/ovac059 36763804

[25] Hatib A, Hassou N, Benani A, Hafid JE, Ennaji MM. 2021. Molecular detection of rotavirus in mollusks from the oued el maleh estuary of mohammedia, Morocco. J Pure Appl Microbiol 15:2358–2366. doi:10.22207/JPAM.15.4.60

[26] Hernroth BE, Conden-Hansson AC, Rehnstam-Holm AS, Girones R, Allard AK. 2002. Environmental factors influencing human viral pathogens and their potential indicator organisms in the blue mussel, Mytilus edulis: the first scandinavian report. Appl Environ Microbiol 68:4523–4533. doi:10.1128/AEM.68.9.4523-4533.2002 12200309 PMC124092

[27] Costafreda MI, Bosch A, Pintó RM. 2006. Development, evaluation, and standardization of a real-time TaqMan reverse transcription-PCR assay for quantification of hepatitis A virus in clinical and shellfish samples. Appl Environ Microbiol 72:3846–3855. doi:10.1128/AEM.02660-05 16751488 PMC1489592

[28] da Silva AK, Le Saux J-C, Parnaudeau S, Pommepuy M, Elimelech M, Le Guyader FS. 2007. Evaluation of removal of noroviruses during wastewater treatment, using real-time reverse transcription-PCR: different behaviors of genogroups I and II. Appl Environ Microbiol 73:7891–7897. doi:10.1128/AEM.01428-07 17933913 PMC2168159

[29] Kageyama T, Kojima S, Shinohara M, Uchida K, Fukushi S, Hoshino FB, Takeda N, Katayama K. 2003. Broadly reactive and highly sensitive assay for Norwalk-like viruses based on real-time quantitative reverse transcription-PCR. J Clin Microbiol 41:1548–1557. doi:10.1128/JCM.41.4.1548-1557.2003 12682144 PMC153860

[30] Loisy F, Atmar RL, Guillon P, Le Cann P, Pommepuy M, Le Guyader FS. 2005. Real-time RT-PCR for norovirus screening in shellfish. J Virol Methods 123:1–7. doi:10.1016/j.jviromet.2004.08.023 15582692

[31] Svraka S, Duizer E, Vennema H, de Bruin E, van der Veer B, Dorresteijn B, Koopmans M. 2007. Etiological role of viruses in outbreaks of acute gastroenteritis in The Netherlands from 1994 through 2005. J Clin Microbiol 45:1389–1394. doi:10.1128/JCM.02305-06 17360839 PMC1865895

[32] Pang XL, Lee B, Boroumand N, Leblanc B, Preiksaitis JK, Yu Ip CC. 2004. Increased detection of rotavirus using a real time reverse transcription-polymerase chain reaction (RT-PCR) assay in stool specimens from children with diarrhea. J Med Virol 72:496–501. doi:10.1002/jmv.20009 14748075

[33] Zeng SQ, Halkosalo A, Salminen M, Szakal ED, Puustinen L, Vesikari T. 2008. One-step quantitative RT-PCR for the detection of rotavirus in acute gastroenteritis. J Virol Methods 153:238–240. doi:10.1016/j.jviromet.2008.08.004 18765254

[34] Ye XY, Ming X, Zhang YL, Xiao WQ, Huang XN, Cao YG, Gu KD. 2012. Real-time PCR detection of enteric viruses in source water and treated drinking water in Wuhan, China. Curr Microbiol 65:244–253. doi:10.1007/s00284-012-0152-1 22645016

[35] Rashed MK, El-Senousy WM, Sayed E, AlKhazindar M. 2022. Infectious pepper mild mottle virus and human adenoviruses as viral indices in sewage and water samples. Food Environ Virol 14:246–257. doi:10.1007/s12560-022-09525-0 35713790 PMC9458564

[36] A. Gad M, K. Allayeh A, M. Elmahdy E, N.F. Shaheen M, M. Rizk N, Z. Al-Herrawy A, R. Saleh FE, A. Marouf M. 2019. Genotyping and interaction-reality of Acanthamoeba, enteric adenovirus and rotavirus in drinking water, Egypt. Egypt J of Aquatic Biolo and Fish 23:65–79. doi:10.21608/ejabf.2019.29299

[37] Ahmed W, Gyawali P, Toze S. 2016. Evaluation of glass wool filters and hollow-fiber ultrafiltration concentration methods for qpcr detection of human adenoviruses and polyomaviruses in river water. Water Air Soil Pollut 227. doi:10.1007/s11270-016-3026-5 PMC7089043 32214527

[38] Miura T, Kadoya S-S, Miura Y, Takino H, Akiba M, Sano D, Masuda T. 2024. Pepper mild mottle virus intended for use as a process indicator for drinking water treatment: present forms and quantitative relations to norovirus and rotavirus in surface water. Water Res 257:121713. doi:10.1016/j.watres.2024.121713 38733963

[39] Giammanco GM, Bonura F, Urone N, Purpari G, Cuccia M, Pepe A, Li Muli S, Cappa V, Saglimbene C, Mandolfo G, Marino A, Guercio A, Di Bartolo I, De Grazia S. 2018. Waterborne Norovirus outbreak at a seaside resort likely originating from municipal water distribution system failure. Epidemiol Infect 146:879–887. doi:10.1017/S095026881800081X 29633676 PMC9184946

[40] Mortari A, Kolling D, Sobral D, Kist A, De Dea Lindner J, Fongaro G, Miotto M. 2023. Norovirus and rotavirus in surface, malacoculture, and human consumption water in Santa Catarina State, Brazil. J Water Health 21:35–46. doi:10.2166/wh.2022.188 36705496

[41] Steyer A, Torkar KG, Gutiérrez-Aguirre I, Poljšak-Prijatelj M. 2011. High prevalence of enteric viruses in untreated individual drinking water sources and surface water in Slovenia. Int J Hyg Environ Health 214:392–398. doi:10.1016/j.ijheh.2011.05.006 21665537

[42] Gibson KE, Opryszko MC, Schissler JT, Guo Y, Schwab KJ. 2011. Evaluation of human enteric viruses in surface water and drinking water resources in southern Ghana. Am J Trop Med Hyg 84:20–29. doi:10.4269/ajtmh.2011.10-0389 21212196 PMC3005515

[43] El-Senousy WM, Rashed MK, Kamel MA, Hasan SF. 2023. Human rotavirus genome copies and infectious units in water and wastewater in Egypt. Egypt J Chem 0:0–0. doi:10.21608/ejchem.2023.225241.8344

[44] Kittigul L, Pombubpa K. 2021. Rotavirus surveillance in tap water, recycled water, and sewage sludge in Thailand: a longitudinal study, 2007-2018. Food Environ Virol 13:53–63. doi:10.1007/s12560-020-09450-0 33128701

[45] Haramoto E, Kitajima M, Hata A, Torrey JR, Masago Y, Sano D, Katayama H. 2018. A review on recent progress in the detection methods and prevalence of human enteric viruses in water. Water Res 135:168–186. doi:10.1016/j.watres.2018.02.004 29471200

[46] Corsi SR, Borchardt MA, Spencer SK, Hughes PE, Baldwin AK. 2014. Human and bovine viruses in the Milwaukee River watershed: hydrologically relevant representation and relations with environmental variables. Science of The Total Environment 490:849–860. doi:10.1016/j.scitotenv.2014.05.072 24908645 PMC7125695

[47] Opere WM, John M, Ombori O. 2020. Occurrence of enteric viruses in surface water and the relationship with changes in season and physical water quality dynamics. Adv Virol 2020:9062041. doi:10.1155/2020/9062041 32695168 PMC7354635

[48] Barbosa LCG, Lima FS, Silva PAN da, Bordoni GP, Scalize PS, Vieira JDG, Carneiro LC. 2023. Association among the presence of rotavirus Group A and types of sources located in rural communities. Water (Basel) 15:1763. doi:10.3390/w15091763

[49] Lambertini E, Spencer SK, Bertz PD, Loge FJ, Kieke BA, Borchardt MA. 2008. Concentration of enteroviruses, adenoviruses, and noroviruses from drinking water by use of glass wool filters. Appl Environ Microbiol 74:2990–2996. doi:10.1128/AEM.02246-07 18359827 PMC2394941

[50] Berrouch S, Escotte-Binet S, Biary A, Nast E, Laaouidi Y, Aubert D, Maarouf A, Harrak R, Villena I, Hafid J. 2023. Investigation of the presence of Toxoplasma gondii, Giardia duodenalis, and Cryptosporidium spp. in drinking waters in the region of Marrakech, Morocco. J Food Prot 86:100112. doi:10.1016/j.jfp.2023.100112 37286083

[51] Alkathiri A, Eifan S, Hanif A, Nour I, Al-Anazi AE, Maniah K, Alotaibi R, Alharbi Y. 2023. Human adenovirus detection and genetic characterization in irrigation water from the riyadh region, Saudi Arabia. Water (Basel) 15:3318. doi:10.3390/w15183318

[52] Cioffi B, Monini M, Salamone M, Pellicanò R, Di Bartolo I, Guida M, La Rosa G, Fusco G. 2020. Environmental surveillance of human enteric viruses in wastewaters, groundwater, surface water and sediments of Campania Region. Regional Studies in Marine Science 38:101368. doi:10.1016/j.rsma.2020.101368

[53] Bertrand I, Schijven JF, Sánchez G, Wyn-Jones P, Ottoson J, Morin T, Muscillo M, Verani M, Nasser A, de Roda Husman AM, Myrmel M, Sellwood J, Cook N, Gantzer C. 2012. The impact of temperature on the inactivation of enteric viruses in food and water: a review. J Appl Microbiol 112:1059–1074. doi:10.1111/j.1365-2672.2012.05267.x 22380614

[54] Meng Z-D, Birch C, Heath R, Gust I. 1987. Physicochemical stability and inactivation of human and simian rotaviruses. Appl Environ Microbiol 53:727–730. doi:10.1128/aem.53.4.727-730.1987 3034154 PMC203745

[55] Pesavento JB, Crawford SE, Roberts E, Estes MK, Prasad BVV. 2005. pH-induced conformational change of the rotavirus VP4 spike: implications for cell entry and antibody neutralization. J Virol 79:8572–8580. doi:10.1128/JVI.79.13.8572-8580.2005 15956598 PMC1143764

[56] Sanchez G, Joosten H, Meyer R, Beuret C. 2005. Presence of norovirus sequences in bottled waters is questionable. Appl Environ Microbiol 71:2203–2205. doi:10.1128/AEM.71.4.2203-2205.2005 15812061 PMC1082572

[57] Samandoulgou I, Hammami R, Morales Rayas R, Fliss I, Jean J. 2015. Stability of secondary and tertiary structures of virus-like particles representing noroviruses: effects of pH, ionic strength, and temperature and implications for adhesion to surfaces. Appl Environ Microbiol 81:7680–7686. doi:10.1128/AEM.01278-15 26296729 PMC4616957

[58] Cuellar JL, Meinhoevel F, Hoehne M, Donath E. 2010. Size and mechanical stability of norovirus capsids depend on pH: a nanoindentation study. J Gen Virol 91:2449–2456. doi:10.1099/vir.0.021212-0 20592107

[59] Rexroad J, Evans RK, Middaugh CR. 2006. Effect of pH and ionic strength on the physical stability of adenovirus type 5. J Pharm Sci 95:237–247. doi:10.1002/jps.20496 16372304

[60] Silva AK, Estes MK, Emilmelech M, Kavanagh OV. 2014. Virus-like particles demonstrate differing responses to solution. Environ Sci Technol 45:520–526. doi:10.1021/es102368d PMC3914881 21121659

[61] Jiménez-Zaragoza M, Yubero MP, Martín-Forero E, Castón JR, Reguera D, Luque D, de Pablo PJ, Rodríguez JM. 2018. Biophysical properties of single rotavirus particles account for the functions of protein shells in a multilayered virus. Elife 7:1–23. doi:10.7554/eLife.37295 PMC6133545 30201094

[62] Favier AL, Burmeister WP, Chroboczek J. 2004. Unique physicochemical properties of human enteric Ad41 responsible for its survival and replication in the gastrointestinal tract. Virology (Auckl) 322:93–104. doi:10.1016/j.virol.2004.01.020 PMC7172780 15063120

[63] Sinclair RG, Jones EL, Gerba CP. 2009. Viruses in recreational water-borne disease outbreaks: a review. J Appl Microbiol 107:1769–1780. doi:10.1111/j.1365-2672.2009.04367.x 19486213

[64] Kang L-H, Oh S, Park J-W, Won Y-J, Ryu S, Paik S-Y. 2013. Simultaneous detection of waterborne viruses by multiplex real-time PCR. J Microbiol 51:671–675. doi:10.1007/s12275-013-3199-1 24037661

[65] La Rosa G, Pourshaban M, Iaconelli M, Muscillo M. 2010. Quantitative real-time PCR of enteric viruses in influent and effluent samples from wastewater treatment plants in Italy. Ann Ist Super Sanita 46:266–273. doi:10.4415/ANN_10_03_07 20847459

[66] Bosch A, Sánchez G, Abbaszadegan M, Carducci A, Guix S, Le Guyader FS, Netshikweta R, Pintó RM, van der Poel WHM, Rutjes S, Sano D, Taylor MB, van Zyl WB, Rodríguez-Lázaro D, Kovač K, Sellwood J. 2011. Analytical methods for virus detection in water and food. Food Anal Methods 4:4–12. doi:10.1007/s12161-010-9161-5

[67] Kishida N, Noda N, Haramoto E, Kawaharasaki M, Akiba M, Sekiguchi Y. 2014. Quantitative detection of human enteric adenoviruses in river water by microfluidic digital polymerase chain reaction. Water Sci Technol 70:555–560. doi:10.2166/wst.2014.262 25098888

[68] Reynolds KA, Gerba CP, Pepper IL. 1996. Detection of infectious enteroviruses by an integrated cell culture-PCR procedure. Appl Environ Microbiol 62:1424–1427. doi:10.1128/aem.62.4.1424-1427.1996 8919804 PMC167909

[69] Chapron CD, Ballester NA, Fontaine JH, Frades CN, Margolin AB. 2000. Detection of astroviruses, enteroviruses, and adenovirus types 40 and 41 in surface waters collected and evaluated by the information collection rule and an integrated cell culture-nested PCR procedure. Appl Environ Microbiol 66:2520–2525. doi:10.1128/AEM.66.6.2520-2525.2000 10831432 PMC110573

[70] Li D, Gu AZ, Yang W, He M, Hu X, Shi H-C. 2010. An integrated cell culture and reverse transcription quantitative PCR assay for detection of infectious rotaviruses in environmental waters. J Microbiol Methods 82:59–63. doi:10.1016/j.mimet.2010.04.003 20399813

[71] Parshionikar S, Laseke I, Fout GS. 2010. Use of propidium monoazide in reverse transcriptase PCR to distinguish between infectious and noninfectious enteric viruses in water samples. Appl Environ Microbiol 76:4318–4326. doi:10.1128/AEM.02800-09 20472736 PMC2897418

